# Diffusion Maps for Multimodal Registration

**DOI:** 10.3390/s140610562

**Published:** 2014-06-16

**Authors:** Gemma Piella

**Affiliations:** Department of Information & Communication Technologies, Universitat Pompeu Fabra, Barcelona 08018, Spain; E-Mail: gemma.piella@upf.edu; Tel.: +34-93-542-1348

**Keywords:** diffusion maps, spectral geometry, diffusion distance, multimodal registration

## Abstract

Multimodal image registration is a difficult task, due to the significant intensity variations between the images. A common approach is to use sophisticated similarity measures, such as mutual information, that are robust to those intensity variations. However, these similarity measures are computationally expensive and, moreover, often fail to capture the geometry and the associated dynamics linked with the images. Another approach is the transformation of the images into a common space where modalities can be directly compared. Within this approach, we propose to register multimodal images by using diffusion maps to describe the geometric and spectral properties of the data. Through diffusion maps, the multimodal data is transformed into a new set of canonical coordinates that reflect its geometry uniformly across modalities, so that meaningful correspondences can be established between them. Images in this new representation can then be registered using a simple Euclidean distance as a similarity measure. Registration accuracy was evaluated on both real and simulated brain images with known ground-truth for both rigid and non-rigid registration. Results showed that the proposed approach achieved higher accuracy than the conventional approach using mutual information.

## 1. Introduction

Feature modeling, as well as dimensionality reduction for image representation is important in fields, such as image analysis and computer vision. In particular, when dealing with multimodal imaging, a main challenge is to obtain a unified representation of the heterogeneous image data, so that meaningful comparisons and effective combinations can be performed.

In this paper, we propose to apply diffusion maps [1] to represent heterogeneous images for multimodal medical image registration. The motivation for using diffusion maps comes from the fact that the resulting embedding captures the intrinsic geometry of the underlying manifold independently of the sampling density and image modality.

**Figure 1.**
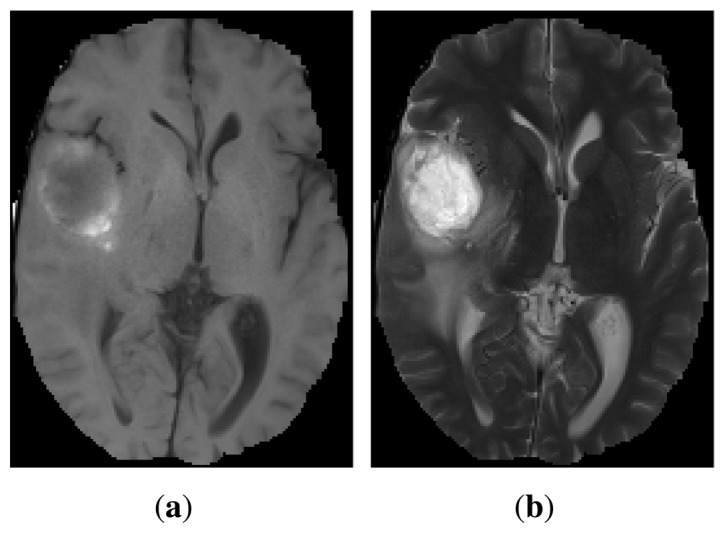
Example of multimodal images: T1 MR (**a**) and T2 MR (**b**) scans of a brain with a tumor.

The purpose of multimodal image registration is to identify the geometric transformation that maps the coordinate system of one modality into another. Multimodal images have significant variations in their intensities, which makes it difficult to capture their structural similarities, thus increasing the difficulty of achieving accurate registration. As an illustration, Figure 1 shows corresponding slices of 3D multimodal images of a human head. While a T1 magnetic resonance (MR) image shows better anatomical detail (Figure 1a), T2 MR highlights pathological changes better (Figure 1b).

Image registration methods can be broadly divided into feature-based and intensity-based techniques [2]. Feature-based methods rely on the extraction and matching of image features, such as points, contours or surfaces. Such methods are often more efficient to compute, but they are usually not effective for multimodal image registration, because the great variation in the intensity values makes it difficult to find enough accurately corresponding features. Intensity-based methods, in contrast, use the intensity values to measure the similarity between corresponding images. For multimodal registration, the current standard is to use mutual information (MI) as the intensity-based image similarity measure due to its ability to handle large intensity variations. However, MI is computationally expensive, because it requires the estimation of probability density functions based on joint histograms. This is further aggravated when jointly analyzing several images.

Structural image representations have gained great interest for multimodal registration. They rely on the assumption that similar internal self-similarities exist across modalities, and hence, once the images are transformed into such representations, efficient monomodal similarity measures (e.g., Euclidean distance) can be used. For example, local gradient orientation has been employed to find correspondences between images [3,4]. However, gradient estimation is problematic for points where more than two regions meet [5]. A more robust approach is the use of local entropy [6] or the multidimensional representation based on local patch distances [7–9]. Nonetheless, all of these approaches rely only on local neighborhoods, and moreover, the local self-similarity pattern itself can be significantly different between corresponding points in the images [10]. Furthermore, not all of these approaches are invariant to rigid deformations.

**Figure 2.**
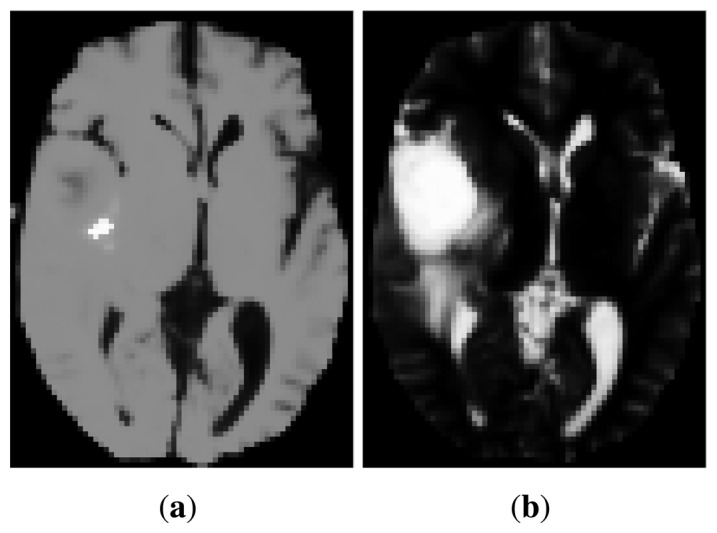
Example of Laplacian eigenmap embeddings for images in Figure 1: Laplacian images for T1 (**a**) and for T2 (**b**). They were obtained by taking the first eigenvector of the graph Laplacian of the original images.

Recently, manifold learning techniques have begun to be applied for image registration [11], although most of the reported methods deal with monomodal data. The most common approach is to apply manifold learning to a population of monomodal images to extract information on the neighborhood structure of the data set (*i.e.*, which images are close to each other) and, hence, confine the search of anatomically plausible transformations [12,13]. There are also a few works where a manifold learning method has been applied to each individual image separately in order to obtain a new representation, which can help in finding image correspondences. For example, Xu *et al.* [14,15] use Isomap or PCA to extract features, which are then passed to a neural network, whose outputs are the affine transformation parameters. Furthermore, in the context of rigid (but multimodal) registration, Wachinger *et al.* [6] employ Laplacian eigenmaps to generate a new set of features, which allows the use of monomodal similarity measures. This is justified because the Laplacian eigenmap can be considered a structural image descriptor, and hence, its appearance across modalities is similar. Nonetheless, there are cases for which the large intensity variations across the original images result in too different eigenmaps preventing from direct comparison. This is illustrated in Figure 2a,b. Other drawbacks are that Laplacian eigenmaps heavily depend on the density of the samples [16] and that an additional step of manifold alignment is required to ensure that the representations of image intensities are comparable across manifolds [6].

Closely related to manifold learning and structural image representations are spectral decomposition methods. Such methods exploit both the local and global structure of the data in the form of distance measures and a kernel, respectively. They have been used extensively in computer vision for locating correspondences between feature point sets [17–20]. The strategy is to embed the point sets into a common eigenspace through an eigendecomposition of a point affinity matrix and to find correspondences by the closest point matching in this space. Spectral methods, however, have been hardly used for dense image registration. Recently, Lombaert *et al.* [21] extended the log demon registration framework [22] to include spectral representations in the nearest-neighbor searches of finding point correspondences between monomodal images. As in [6], they use Laplacian eigenmaps for the spectral embedding. The resulting scheme is shown to capture large and complex deformations in monomodal image registration. This approach would not work on multimodal registration; on the one hand, because log demons cannot deal with multiple modalities, and on the other hand, because of the aforementioned drawbacks of using the Laplacian eigenmaps directly as structural representations.

We propose to use diffusion mappings [1] to obtain a unified representation of multimodal images corresponding to the same underlying object (e.g., the same organ in medical imaging). Diffusion mapping is a Laplacian-based spectral embedding method based on defining a random walk on the graph of the data. It constructs a discrete density-independent approximation of the Laplace-Beltrami operator, providing an embedding that captures the intrinsic geometry while taking into account the density of samples and the fact that data structures may occur at different scales. Diffusion maps have been shown to robustly represent image features [1,23]. Therefore, we propose to estimate local deformation between images by matching their corresponding diffusion maps. In this way, multimodal registration is performed in the Laplace-Beltrami spectral domain instead of the usual image spatial domain. In addition, since the Laplace-Beltrami spectrum is an isometric invariant, diffusion map embeddings are invariant under isometric deformations, such as rotations and translations [24]. While diffusion maps have been used for various applications, they have not been used, to the best of our knowledge, for multimodal image registration.

The remainder of this paper is structured as follows: The next section reviews diffusion map embedding and describes how it can be used for multimodal registration. Details of the algorithm implementation are presented, which reduce the computational complexity of the embedding construction. Section 3 shows an evaluation of robustness and the accuracy of the proposed method for both rigid and non-rigid registration. Finally, results are discussed and future work directions are given.

## 2. Methods

We use diffusion maps to transform the multimodal data into a new set of canonical coordinates that reflect its geometry uniformly across modalities and different parameters (e.g., time, acquisition view or subject). That is, we look for structure invariants in the data in order to be able to establish meaningful correspondences between different realizations of the data.

In this section, we first review the diffusion framework and its extension to changing data and then explain how we use it for multimodal image registration.

### 2.1. The Diffusion Framework

The diffusion maps framework introduced in [1] gives a multiscale organization of the data revealing different geometric structures at different scales. At each scale, diffusion mapping embeds the data into a particular Euclidean space in which the usual Euclidean distance corresponds to the diffusion distance on the data at that scale. Since diffusion maps are able to capture the main structures of the data in a few dimensions, the embedded Euclidean space can be truncated to a lower dimensional space, hence achieving dimensionality reduction.

Let $\chi ={\left\{x_{i}\right\}}_{i=1}^{n}$, *x_i_* ∈ **ℝ***^d^*, be a (possibly high-dimensional) set of data points. A weighted symmetric graph is constructed in which each data point *x_i_* corresponds to a node, and the weights of the edges connecting two nodes are a measure of the similarity between them. A popular choice is to weight the edge between data points *x_i_* and *x_j_* by using a Gaussian kernel, *w*(*x_i_*,*x_j_*) = *exp*(− ‖*x_i_* − *x_j_*‖^2^/2*σ*^2^), where *σ* > 0 is the kernel bandwidth. The normalized kernel

$$p(x_{i},x_{j})=\frac{w(x_{i},x_{j})}{\sum_{x_{j}\in \chi }w(x_{i},x_{j})}$$

encodes the probability of transition from *x_i_* to *x_j_*, measuring the influence of these points with the rest of the graph. Therefore, matrix *P* ∈ *R^n^*^×^*^n^* with *p*(·,·) as its entries is a Markov transition matrix. Taking powers of *P* amounts to running the Markov chain forward in time, revealing geometric structures of *χ* at different scales. That is, if *P^t^* is the *t*-th power of matrix *P*, then its corresponding kernel *P_t_*(*x_i_*, *x_j_*) represents the probability of transition from *x_i_* to *x_j_* in *t* time steps. Increasing *t* corresponds to propagating the local influence of each point with its neighbors, which means that *t* can be seen as a scale parameter.

Using spectral theory, it can be shown that kernel *p_t_* has the following eigendecomposition:

(1) $$p_{t}(x_{i},x_{j})=\sum_{k\geq 0}\lambda_{k}^{t}\psi_{k}(x_{i})\phi_{k}(x_{j})$$

where {λ*_k_*} is the decreasing eigenspectrum of *P* and {*ψ_k_*}, {*ϕ_k_*} are the corresponding biorthogonal right and left eigenvectors. One can then define a metric on the data, known as the diffusion distance, that measures the amount of connectivity between any two points *x_i_* and *x_j_*:

(2) $$D_{t}^{2}(x_{i},x_{j})=\sum_{x_{k}\in \chi }\frac{{\left(p_{t}(x_{i},x_{k})-p_{t}(x_{k},x_{j})\right)}^{2}}{\phi_{0}(x_{k})}$$

Combining Equations (1) and (2), together with biorthogonality properties, the diffusion distance can also be expressed as:

(3) $$D_{t}^{2}(x_{i},x_{j})=\sum_{k\geq 1}\lambda_{k}^{2t}{\left(\psi_{k}(x_{i})-\psi_{k}(x_{j})\right)}^{2}$$

where *ψ*_0_ has been omitted in the sum above, because it is a constant vector. Hence, {*λ_k_ψ_k_*} can be used as a new set of coordinates for the set *χ*. The diffusion map is therefore defined as the embedding of the data set *χ* into the Euclidean space **ℝ***^l^*:

$${\begin{array}{cc} \Psi :\chi \mapsto {\mathbb{R}}^{l}, & \Psi (x_{i})=(\lambda_{1}\psi_{1}(x_{i}),\ldots ,\lambda_{l}\psi_{l}(x_{i})) \end{array}}^{T}$$

With this definition, the diffusion distance between *x_i_* and *x_j_* ∈ *χ* is equal to the Euclidean distance between diffusion coordinates Ψ(*x_i_*) and Ψ(*x_j_*), when using all *l* = *n* − 1 eigenvectors:

$$D_{t}^{2}\left(x_{i},x_{j}\right)={\left\|\Psi \left(x_{i}\right)-\Psi \left(x_{j}\right)\right\|}^{2}$$

Furthermore, because of the spectrum decay of the eigenvalues, the diffusion distance can be approximated by the embedded samples in low dimensions (*i.e.*, setting *l* to a small value).

As proposed in [23], a completely density invariant Markov chain can be built by re-weighting the kernel as:

$$\tilde{w}(x_{i},x_{j})=\frac{w(x_{i},x_{j})}{\sum_{x_{j}\in \chi }w(x_{i},x_{j})\sum_{x_{i}\in \chi }w(x_{i},x_{j})}$$

Then, the entries of the transition probability matrix become:

$$p(x_{i},x_{j})=\frac{\tilde{w}(x_{i},x_{j})}{\sum_{x_{j}\in \chi }\tilde{w}(x_{i},x_{j})}$$

In this way, one can recover the geometry of the manifold independently of the data density Moreover, it can be shown that, assuming that the points of *χ* lie in a manifold of **ℝ***^d^*, *P* generates a diffusion matrix that approximates the Laplace-Beltrami operator [1]. The Euclidean distance between two samples in the embedded coordinate system (*i.e.*, the diffusion distance) is dependent not only upon the original pairwise similarity between them, but also upon all the pairwise similarities that each sample has with the remainder of the population. This makes diffusion distance a more robust metric than individual pairwise measures of similarity, which can be susceptible to noise.

### 2.2. Diffusion Maps for Changing Data

The diffusion mapping provides a new representation of data sets reflecting their intrinsic geometry. This new representation is based on the neighbor relationship between points (*i.e.*, geometry information) and not on their original feature representation (e.g., pixel intensities). As a consequence, when dealing with various data sets that need to be put into correspondence, it can be more effective to compare their embeddings instead of the original data sets.

However, in some scenarios, a direct comparison between embeddings from data obtained in different conditions may not provide meaningful connections. This issue is addressed in [25], where the authors generalize diffusion maps for data sets that evolve over time or that change depending on some input parameters (e.g., data obtained under different conditions or sensors). The extension consists mainly of a mapping between the embeddings of the different data sets.

Let 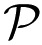 be some parameter space, *χ^α^* the data that depends on parameter *α* ∈ 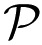 and $\Psi^{\alpha }\left(‥\right)={\left(\lambda_{k}^{\alpha }\psi_{k}^{\alpha }\left(‥\right)\right)}_{k\geq 1}$ its corresponding diffusion map (where we have dropped subindex *t* for the simplicity of notation). For different parameters *α*_1_,*α*_2_ ∈ 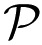, diffusion mapping may take *χ^α^*^1^, *χ^α^*^2^ into different Euclidean spaces, thus meaning that the standard Euclidean distance between the elements of these spaces is not meaningful. Since ${\left\{\psi_{k}^{\alpha }\right\}}_{k\geq 1}$ is a basis for the embedding of *χ^α^*, one can use a change of basis operator to map one embedding into another, *i.e.*,

(4) $$O^{\alpha_{2}\to \alpha_{1}}\Psi^{\alpha_{2}}={\left(\sum_{j\geq 1}\Psi_{j}^{\alpha_{2}}\langle \psi_{k}^{\alpha_{1}},\psi_{k}^{\alpha_{2}}\rangle \right)}_{k\geq 1}$$

where (·, ·) denotes the inner product and Ψ*_j_* = *λ_j_ψ_j_*. In this way, Ψ*^α^*^1^ and *O^α^*^2^*^→α^*^1^ Ψ*^α^*^2^ bring their corresponding data sets *χ^α^*^1^ and *χ^α^*^2^ to the same Euclidean space. This generalizes the diffusion distance from within a data set to between data sets and allows computing distances between any *x_i_* ∈ *χ^α^*^1^ and ${x^{\prime }}_{j}\in \chi^{\alpha 2}$.

### 2.3. Application to Multimodal Image Registration

For each parameter *α* (e.g., modality or time), let $I^{\alpha }:\Omega \mapsto {\mathbb{R}}^{m}$ be the corresponding image, where Ω ⊆ **ℝ**^2^ denotes the image domain and *I^α^*(*u*) represents the intensity level at pixel *u* ∈ Ω (or the intensity values within a patch centered at *u*). The associated data *χ^α^* is the set of points ${\left\{x_{i}=\chi^{\alpha }\left(u_{i}\right)=\left(\beta u_{i},I^{\alpha }\left(u_{i}\right)\right)\right\}}_{i=1}^{n}$, *u_i_* ∈ Ω, with *β* being a constant controlling the influence of spatial locality.

For each *χ^α^*, we compute the Gaussian kernel with bandwidth *σ* selected as the median pairwise distance between data points, as proposed in [26]:

$$\sigma^{2}=\text{median}\left\{{\left\|x_{i}-x_{j}\right\|}^{2}\right\}i,j=1,\ldots ,n$$

where ‖ · ‖ is the Euclidean distance. For the scale parameter *t*, which determines the strength of the diffusion, we combine the information at all scales into one single description. Hence, instead of using a fixed scale *t* to compute *D_t_*, we compute the diffusion map by considering diffusion distances *D_t_* at all scales:

(5) $$\begin{array}{cc} {\tilde{D}}^{2}\left(x_{i},x_{j}\right) & =\sum_{t=1}^{\infty }D_{t}^{2}\left(x_{i},x_{j}\right)\\ & =\sum_{t=1}^{\infty }\sum_{k\geq 1}\lambda_{k}^{2t}{\left(\psi_{k}\left(x_{i}\right)-\psi_{k}\left(x_{j}\right)\right)}^{2}\\ & =\sum_{k\geq 1}{\tilde{\lambda }}_{k}^{2t}{\left(\psi_{k}\left(x_{i}\right)-\psi_{k}\left(x_{j}\right)\right)}^{2} \end{array}$$

where ${\tilde{\lambda }}^{2}=\overset{\infty }{\underset{t=1}{\text{∑}}}\lambda^{2t}=\frac{\lambda^{2}}{1-\lambda^{2}}.$

For ensuring meaningful comparisons between images derived from different conditions, we map each embedding Ψ*^α^* into a common Euclidean space using Equation (4). From this new diffusion map embedding, we build an image by assigning to each pixel a vector with the *l* firsts coordinates of the embedding. We refer to this image as the diffusion-map image. In an abuse of notation, we use the same letter Ψ for both the embedding and its image representation.

The diffusion-map images are the input images for a registration algorithm. The goal of image registration is to find a suitable transformation *φ* that aligns one image to another. This can be done by minimizing the cost function that measures the distance between the involved images:

$$\underset{\varphi }{\min}D^{\Psi }\left(\Psi^{\gamma ,\alpha },\Psi^{\gamma ,\alpha \prime }\circ \varphi \right)$$

where Ψ^γ,α^ = *O^α^*^→γ^Ψ*^α^*, Ψ^γ^,*^α^*′ = *O^α^*′^→γ^Ψ*^α^*′ are the diffusion-map images (in the Euclidean space spanned by Ψ^γ^), and 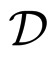
^Ψ^ : **ℝ***^l^* × **ℝ***^l^* → **ℝ** is chosen to be the Euclidean distance. Since the Euclidean distance on the diffusion-map images is equivalent (up to some accuracy) to the diffusion distance on the original image:

(6) $$\begin{array}{cc} D^{\Psi }{\left(\Psi^{\gamma ,\alpha },\Psi^{\gamma ,\alpha \prime }\circ \varphi \right)}^{2} & =\sum_{i=1}^{n}{\left\|O^{\alpha \to \gamma }\Psi^{\alpha }\left(\chi^{\alpha }\left(u_{i}\right)\right)-O^{\alpha \prime \to \gamma }\Psi^{\alpha \prime }\left(\chi^{\alpha \prime }\left(\varphi \left(u_{i}\right)\right)\right)\right\|}^{2}\\ & ≃\sum_{i=1}^{n}\tilde{D}{\left(\chi^{\alpha }\left(u_{i}\right),\chi^{\alpha \prime }\left(\varphi \left(u_{i}\right)\right)\right)}^{2}≐D^{\chi }{\left(\chi^{\alpha },\chi^{\alpha \prime }\circ \varphi \right)}^{2} \end{array}$$

where *D̃* is the diffusion distance defined in Equation (5) and 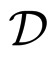
*^χ^* : Ω × Ω → **ℝ** is the global diffusion distance between the images (represented here by the intensity values of pixels and their spatial coordinates).

### 2.4. Implementation

In our implementation, we set *β* = 1 (*i.e., x* = (*u, I^α^*(*u*)) for all *u* ∈ Ω and, to compensate for the different scalings of the intensity values, each image was initially rescaled, so that its range fitted the range of the coordinate values. To reduce the computational cost, the diffusion map was constructed using the coarsest level of a Gaussian pyramid representation of the image. The embedding coordinates were then extended to all samples in the original image using the multiscale Laplacian pyramid [26]. Furthermore, for simplicity, diffusion-map images were constructed using the first (*l* = 1) coordinate (thus, we only deal with scalar diffusion-map images). This was enough for our purposes, since the decay rate of the spectrum was high and *l* = 1 captured the most relevant features.

For the non-rigid registration, the transformation was modeled by a B-spline free form deformation (FFD) with a control point spacing of 4 pixels. A gradient-based optimization strategy was used.

The algorithms were implemented in MATLAB and ran on a Linux machine with a dual quad-core Intel Xeon (1.6 GHz CPU, 4 GB RAM). The average computation time when using the proposed approach for rigid and non-rigid registration was about 5 min (for images of size 181 × 217), the burden of the computation being on the construction of the diffusion maps. In contrast, the average time for rigid and non-rigid registration using mutual information was 2 and 44 min, respectively.

## 3. Experimental Results

We evaluated the proposed framework on both simulated and real data sets obtained from the Simulated Brain Database (BrainWeb [27] and the Brain Tumor Segmentation (BRATS) challenge [28], respectively.

**Figure 3.**
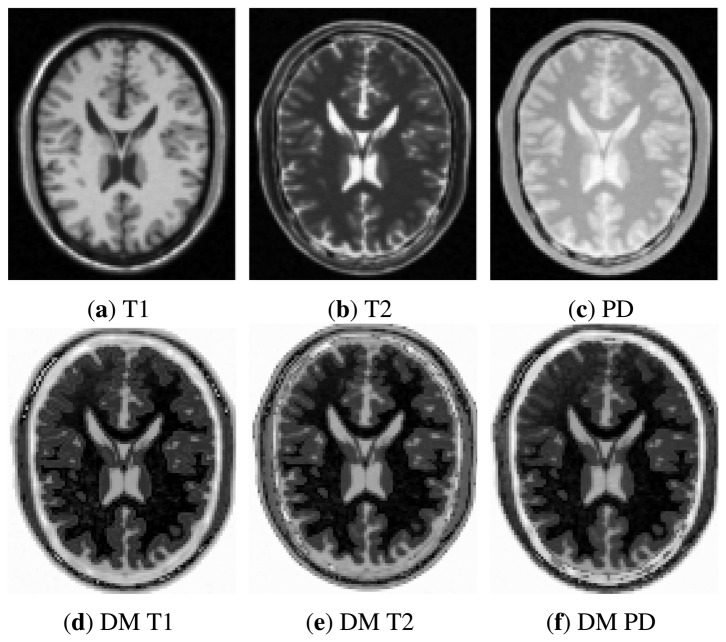
Example of multimodal images from BrainWeb data set **(top row)** and their corresponding diffusion-map images **(bottom row).**

**Figure 4.**
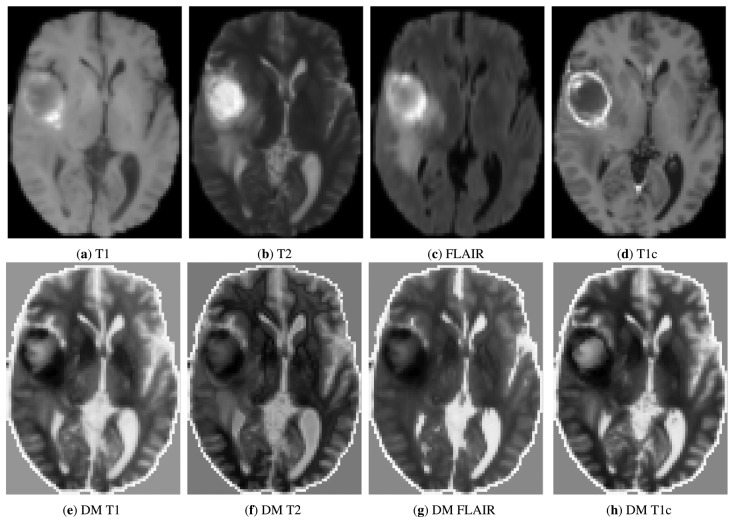
Example of multimodal images from the Brain Tumor Segmentation (BRATS) data set **(top row)** and their corresponding diffusion-map images **(bottom row).**

Images from BrainWeb were simulated with a slice thickness of 1 mm, a noise level of 3% and an intensity non-uniformity of 20%. A total of eight data sets were generated comprising T1-, T2- and proton density (PD)-weighted MR simulated images of the normal brain. The data sets of BRATS comprised T1, T2, FLAIR and post-Gadolinium T1c MR images of 15 patients with glioma (1-mm isotropic resolution). For both databases, the ground-truth alignment was provided. Figures 3 and 4 show axial slices of the original and diffusion-map images.

We distinguished between two cases depending on whether modality is considered or not as a changing parameter of the data. In the first case, each image modality is taken as a different data set, and the purpose is to find the correspondence between pairs of modalities that are not aligned. In the second case, all (co-registered) modalities form a multichannel image, and the purpose is to find the matching between pairs of non-aligned multichannel images.

We assessed the registration accuracy when using as similarity measures the Euclidean distance (L2) on the original images, the mutual information (MI) on the original images and the Euclidean distance on the diffusion-map images obtained as described in Section 2.3.

### 3.1. Separate Modality Analysis

In this section, we apply the proposed methodology to each image modality independently of the others. Each modality is considered as a different parameter *α*. Hence, *χ^α^* corresponds to a single image modality with data points *x* = (*u,I^α^*(*u*)) ∈ **ℝ**^3^ being the concatenation of the position and (single-value) intensity of each pixel. For each modality, we computed its diffusion-map image. We chose the common Euclidean space to be the one corresponding to the T1-weighted modality.

First, we performed pairwise rigid registration for the various combinations of multimodal images. A total of 100 transformations were randomly generated in the ±10 mm range for each translation axis and the ±10° range for each rotation axis. Tables 1 and 2 show the mean registration error for the BrainWeb and BRATS data sets, respectively. We obtained a significantly lower error with diffusion distance in comparison to MI and L2 on the original images. As expected, using L2 on the original images resulted in a higher error than using MI or the diffusion distance (*i.e.*, L2 on the diffusion-map images).

Second, we considered non-rigid pairwise registration. We deformed one of the two images with five known deformations (maximum displacement of 7.2 mm). These ground-truth transformations were generated randomly by using a linear combination of radial basis functions. Next, we performed the registration with the deformed image as the reference image. The error of the registration was computed as the mean Euclidean difference between the ground-truth deformation and the estimated one. Tables 3 and 4 show the results. Again, the lowest errors were obtained with diffusion distance. Errors using L2 distance on the original images were significantly much higher and were not included in the tables.

**Figure 5.**
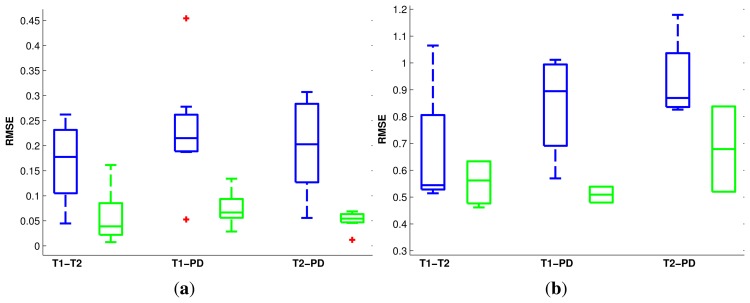
Boxplots of the root mean squared registration errors (in mm) for the BrainWeb data sets. For each pair of modalities, the left-most (blue) boxplots correspond to using MI, while the right-most (green) ones correspond to diffusion distance. Red crosses indicate outliers, **(a)** Rigid registration; **(b)** non-rigid registration.

**Figure 6.**
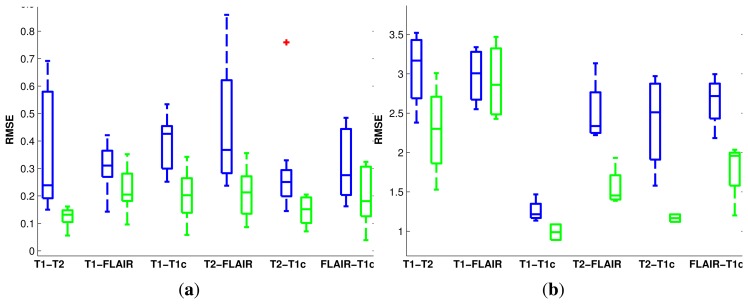
Boxplots of the root mean squared registration errors (in mm) for the BRATS data sets. For each pair of modalities, left-most (blue) boxplots correspond to using MI, while right-most (green) ones correspond to diffusion distance. Red crosses indicate outliers. **(a)** Rigid registration; **(b)** non-rigid registration.

The distribution of errors is summarized in Figures 5 and 6 for both the rigid and non-rigid cases. For the rigid case, we have considered a 1-mm error equal to a 1° error to quantify the translational and rotational displacement from the ground-truth in a single value. In each box, the central mark is the median; the edges of the box are the first and third quartiles, and the whiskers extend to the most extreme data values.

### 3.2. Joint Modality Analysis

In this section, we apply our registration approach to multichannel images in which each pixel is an *m*-dimensional vector, *m* being the number of modalities (*i.e., m* = 3 for the BrainWeb data sets and *m* = 4 for the BRATS ones). Thus, *χ^α^* consists of data points *x* = (*u, I^α^*(*u*)) ∈ **ℝ**^2+^*^m^*. The goal is to perform a unified joint registration using the information of all *m* modalities.

We applied the same deformation schemes as before, and for each multichannel image, a single diffusion-map image encoding the joint spectral geometry was obtained. We chose the common Euclidean space to be the one corresponding to the non-deformed multichannel image.

Registration errors (see Table 5) revealed an increase of at least 14% in accuracy with respect to the separate modality analysis approach.

## 4. Discussion

For both rigid and non-rigid registration, errors were smaller when using the L2 distance on the diffusion-map images than when using standard MI or L2 on the original images. Mean and median registration error are measures of the accuracy of the method, while standard deviation and interquartile ranges are indicators of its robustness. With the original images, the large registration errors obtained for L2 indicate that some registrations failed, while large values of dispersion for MI suggest that some registrations did not converge correctly. Compared to the lower errors and deviations obtained for the diffusion distance, we conclude that registration is more accurate and robust when using diffusion-map images. Furthermore, the better performance of the joint modality approach suggests that using diffusion maps to merge information from different modalities can help in achieving a higher accuracy in registration.

Our approach illustrates the feasibility of dense registering multimodal images. We restricted ourselves to a simple FFD transformation model and applied the proposed scheme to multimodal brain image registration. The framework, however, generalizes to other transformation models (e.g., diffeomorphisms), and it could be useful, also, in other registration scenarios. In particular, diffusion maps can be useful for representing data of the same underlying phenomena, but acquired with different devices or sensors, possibly at various sampling rates and at different times.

As any other spectral method, the core of diffusion maps lies in the construction of a kernel quantifying the connections between the data samples. We chose the Gaussian kernel with the Euclidean distance to capture these connections. This choice was geometrically motivated, since it has locality properties, and furthermore, it is related to the heat kernel for which the Laplace-Beltrami operator is a generator. However, other application-oriented kernels may be used. It remains a challenge how to automatically determine the optimal kernel and its parameters. For example, when using a Gaussian kernel, the choice of the kernel bandwidth *σ* affects the shape and topological properties of the resulting manifold. Setting *σ* too small results in a disconnected graph, where many points are only connected to themselves, while setting *σ* too large results in all points of the graph being connected. Finding the appropriate bandwidth parameter is dependent upon how much local information is needed. As described in Section 2.3, we selected *σ* as proposed in [26]. Alternatively, one could have used an adaptive *σ* as suggested by Zelnik-Manor *et al.* [29]. Furthermore, the Euclidean distance in the kernel could have been replaced by any other metric.

Related to the choice of kernel and metric is the choice of input feature space. We constructed the diffusion maps using as features both the position and intensity of the pixels in the image. An interesting direction to explore would be to use richer features, such as contours and texture descriptors.

Another limitation of our implementation is that we used *l* = 1 to speed up the calculations. The first diffusion coordinates represent the coarse (*i.e.*, low-frequency) intrinsic geometry of the image, while higher ones represent the finer (high-frequency) geometric details. These higher frequency components may reveal important geometric features for registration. It is expected that as the number of retained terms increases, better results can be obtained, although with a significantly higher computational cost.

## 5. Conclusions

We have described a multimodal registration framework that uses diffusion maps for the structural representation of the images to be registered. The new embeddings are all mapped into a common space, allowing one to establish correspondences between them. In this common space, using the Euclidean distance as a similarity measure is equivalent to using the diffusion distance in the space of the original images.

The proposed approach was validated using both synthetic and real brain images. Results showed that the diffusion distance metric improves the registration accuracy and robustness as compared with the classical formulation of MI.

Future work will aim at investigating the use of features, such as textures or contours, when constructing the diffusion maps. We will also investigate the use of parallel computing using GPU accelerators to reduce the computational burden of the spectral decomposition.

## Figures and Tables

**Table 1.** Mean (rigid) registration error for BrainWeb data sets. Rotation errors *r* are in degrees, and translation errors *t* are in mm. PD, proton density; MI, mutual information.

Similarity	T1-T2		T1-PD		T2-PD	
		
	*r*	*t*	*r*	*t*	*r*	*t*
**L2**	62.941	60.250	48.238	53.092	0.294	0.142
**MI**	0.105	0.112	0.175	0.123	0.147	0.107
**Diffusion**	**0.009**	**0.056**	**0.048**	**0.053**	**0.021**	**0.043**

**Table 2.** Mean (rigid) registration error for BRATS data sets. Rotation errors *r* are in degrees, and translation errors *t* are in mm.

Similarity	T1-T2		T1-FLAIR		T1-T1c		T2-FLAIR		T2-T1c		FLAIR-T1c	
					
	*r*	*t*	*r*	*t*	*r*	*t*	*r*	*t*	*r*	*t*	*r*	*t*
**L2**	5.870	0.757	0.705	0.154	0.223	**0.069**	6.008	1.662	8.998	0.628	0.152	**0.095**
**MI**	0.264	0.216	0.278	**0.096**	0.304	0.205	0.360	0.186	0.242	0.139	0.287	0.102
**Diffusion**	**0.033**	**0.093**	**0.157**	**0.107**	**0.162**	**0.069**	**0.155**	**0.079**	**0.096**	**0.084**	**0.111**	0.096

**Table 3.** Mean (non-rigid) registration error for BrainWeb data sets. Errors (mean ± standard deviation) are in mm.

Similarity	T1-T2	T1-PD	T2-PD
**MI**	0.667 ±0.531	0.842 ±0.403	0.936 ± 0.329
**Diffusion**	**0.554 ±0.183**	**0.509 ±0.068**	**0.679 ±0.367**

**Table 4.** Mean (non-rigid) registration error for BRATS data sets. Errors (mean ± standard deviation) are in mm.

Similarity	T1-T2	T1-FLAIR	T1-T1c	T2-FLAIR	T2-T1c	FLAIR-T1c
**MI**	3.054 ±1.004	2.975 ±0.735	1.078 ±0.290	2.506 ±0.849	2.393 ± 1.248	2.654 ±0.683
**Diffusion**	**2.285 ±1.221**	**2.954 ±1.001**	**0.987 ±0.229**	**1.556 ±0.507**	**1.166 ±0.111**	**1.787 ±0.786**

**Table 5.** Mean registration error when using multichannel images. Errors (mean ± standard deviation) are in mm.

Data Set	Rigid	Non-Rigid
**BrainWeb**	0.040 ±0.012	0.401 ±0.104
**BRATS**	0.123 ±0.048	0.844 ± 0.504
